# Knowledge and Practices of Oral Health Care During Pregnancy: A Survey Among Swiss Dentists

**DOI:** 10.3290/j.ohpd.a44682

**Published:** 2020-07-04

**Authors:** Maria Razban, Catherine Giannopoulou

**Affiliations:** a Dentist, Private Practice, Bulle, Switzerland. In fulfillment of requirements for a Doctoral degree, acquired, analysed and interpreted data, critically revised the manuscript and approved the final version.; b Associate Professor, Division of Regenerative Dentistry and Periodontology, University Clinics of Dental Medicine, University of Geneva, Geneva, Switzerland. Study design, analysis and interpretation of the data, wrote the manuscript and approved the final version.

**Keywords:** dentists, education, oral care, pregnancy

## Abstract

**Purpose:**

To evaluate the knowledge and practices of Swiss dentists concerning oral care during pregnancy.

**Materials and Methods:**

A cross-sectional survey was conducted among 200 dentists from the German and French part of Switzerland. The survey consisted of 16 questions which assessed the knowledge, attitudes and barriers faced by dentists regarding dental care during pregnancy.

**Results:**

The majority of dentists agreed that dental care should be part of prenatal care. Overall, good agreement between the French- and German-speaking dentists was found concerning the timing of conducting various dental procedures and the administration of anesthetics and other drugs during pregnancy. Uncertainty was observed regarding the link between periodontal disease and adverse pregnancy outcomes.

**Conclusion:**

The survey reported that Swiss dentists in private practice have the knowledge to provide dental care to pregnant women. However, they all expressed the need for clear guidelines and direction on this important aspect of public health.

Pregnancy is a dynamic physiological state associated with several systemic and local changes due to increased hormonal secretion. The systemic changes may affect the cardiovascular, respiratory, renal and endocrine systems, whereas locally, the oral cavity is one of the main parts of the body where physical changes occur. In recent years, interest in oral health during pregnancy has increased, mainly due to a plethora of studies reporting an association between periodontitis and adverse pregnancy outcomes. In fact, it is the establishment of a chronic low-grade systemic inflammation that has been proposed as the main mechanism linking periodontitis not only with adverse pregnancy outcomes but with several other systemic diseases.^31^ To date, 57 different conditions and diseases have been described as being potentially related to periodontal disease.^28^

The original case-control study by Offenbacher et al^29^ including 124 pregnant women reported that ‘periodontal diseases represent a previously unrecognised and clinically significant risk factor for preterm low birth weight as a consequence of either preterm labor or preterm premature rupture of membranes’. Since then, the association between periodontitis and adverse pregnancy outcomes has been the subject of numerous investigations, however with contradictory results. The consensus report of the Joint EFP/AAP workshop on periodontitis and systemic diseases aimed to review the existing evidence by assembling data from epidemiological studies, studies on the potential biological mechanisms linking the two conditions and from periodontal intervention studies.^35^ The authors reported a modest but statistically significant association between maternal periodontitis and pre-term birth, low birthweight and pre-eclampsia, events that were associated with specific bacterial species. Interventional studies – most of which were performed during the second trimester – reported mixed results: in some studies maternal periodontal treatment reduced the risk of pre-term low birth weight^24,26^ while others contradicted these findings.^27,30^ However, all studies emphasised the key role of oral health in overall health and the importance of providing oral health education and treatment during pregnancy. Other topics that have been addressed in relation to oral health during pregnancy are the timing of conducting different procedures, i.e. endodontic treatment, surgery, radiographs, as well as the safety of drug administration, including anesthetics, antibiotics and non-steroidal anti-inflammatory drugs (NSAIDs) during pregnancy.

In recent years, various surveys have been conducted concerning the knowledge, attitudes, and practices of oral health and dental care during pregnancy. These surveys were addressed to gynaecologists/obstetricians,^3,7,15,17,19,25,33^ dental practitioners,^3,12,13,16,20,22,23,32,33^ dental hygienists,^2,11,36^ and pregnant women^1,4,6,14,38,43^ by means of various questionnaires.

Although some discrepancies were found in the perception of oral health and dental care during pregnancy between the studies, all medical practitioners agreed that there is a need for better education and interprofessional collaboration concerning dental care and the prevention of adverse pregnancy outcomes related to oral health.

To our knowledge, a similar study has never been conducted in Switzerland. We recently conducted a survey among pregnant women attending four public hospitals in Switzerland. Almost half of the participants were aware that a poor oral status may be a risk factor for pregnancy complications; however, the majority of women did not visit their dentist during pregnancy and did not change their oral hygiene habits during this period (unpublished data). The purpose of the present survey was to evaluate the views, knowledge and attitudes of a sample of Swiss dentists on oral health practices for women during pregnancy. Dentists from both the French- and German-speaking parts of Switzerland were asked to participate in order to obtain a representative sample of the whole population.

## Materials and Methods

### Sample and Data Collection

Two hundred dentists practicing in the German- and French-speaking parts of Switzerland were randomly selected to participate in a cross-sectional survey. The questionnaire together with an information sheet was sent by post, and 9 months later to 100 more dentists from both regions in order to acquire a sufficient number of answers.

### Questionnaire

The questionnaire consisted of 16 items, the majority of them derived from existing questionnaires used in similar studies.^15,16^ The first part consisted of questions on age, sex, working area, years since graduation, university at which they received their degree and specialisation certification (general dentistry, orthodontics, periodontics, prosthodontics or maxillofacial surgery). The participants were further asked about the total number of patients they consult per week in their private practice, the number of pregnant women per month, and whether education on oral complications and oral health care during pregnancy was included in their university curriculum. The following questions included items on their knowledge about different oral health conditions during pregnancy, with possible answers being ‘strongly agree’, ‘partly agree’ or ‘disagree’. Furthermore, dentists were asked to indicate the preferred timing for various treatment practices (scaling, root planing, orthodontic treatment, endodontic treatment) as well as the best timing for the administration of anesthetics and other drugs. Finally, the participants were asked to report the main obstacles for providing dental care during pregnancy and whether more education during undergraduate studies and/or continuous education is needed. Questions concerning the timing for treatment practices and drug administration as well as for the barriers in providing dental care during pregnancy could have more than one answer.

### Statistical Analysis

We used descriptive statistics with frequency and percentages to explore the various items addressed to the dentists.

## Results

### Demographics

Two hundred eighty questionnaires were sent between April 2018 and September 2019, and 200 were completed (71% response rate). Responses were received from 58.5% (N=117) male and 41.5% (N=83) female dentists. The mean age was 51 years (range 26-79). As shown in Table 1, the years of experience varied from 1 to 50 years with the majority reporting not having any postgraduate qualification. 80% of the dentists had graduated from a Swiss university and 17% from universities of other European countries; the remaining did not report the country of graduation. The majority of the participants (36.6%) see 40-60 patients per week, followed by those (31%) treating more than 60 patients. 80% of both regions treat between 1 to 3 pregnant women per month. Half of the dentists from both the French and German parts reported having received sufficient information on oral health care during pregnancy (50.5%). However, only 29% stated having information brochures on oral health during pregnancy in their private practice.

**Table 1:** Characteristics of Swiss dentists

	French-speaking n=100	German-speaking n=100	Total n=200
**Gender**			
Female	42 (42%)	41 (41%)	83 (41.5%)
Male	58 (58%)	59 (59%)	117 (58.5%)
**Age range in years**	26-79	28-72	26-79
**Years of experience**	1-47	2-50	1-50
**Number of patients per week**			
20-40 40-60 >60	26 (26%) 29 (29%) 32 (32%)	25 (25% 44 (44%) 30 (30%)	51 (25.5%) 73 (36.5%) 62 (31%)
**Number of pregnant women per month**			
1-3 3-5	75 (75%) 12 (12%)	83 (83%) 6 (6%)	158 (79%) 18 (9%)
**Education/training on oral health care during pregnancy**			
No	0 (0%)	5 (5%)	5 (2,5%)
Yes, but insufficient	54 (54%)	41 (41%)	95 (47,5%)
Yes, sufficient	46 (46%)	55 (55%)	101 (50,5%)
**Information/brochure on oral health during pregnancy in your daily practice**			
Yes	23 (23%)	37 (37%)	60 (30%)
No	77 (77%)	63 (63%)	140 (70%)

The dentists’ knowledge and attitudes on several perinatal oral health items is shown in Table 2. The majority of dentists (61% of the French-speaking and 69% of the German-speaking) disagreed that it is not worthwhile to discuss the link between periodontal disease and adverse pregnancy outcomes with their patients because of insufficient data. All dentists agreed that pregnant women should receive preventive care before and during pregnancy, whereas only a minority (12% for French- and German-speaking dentists alike) reported that pregnant women should receive only emergency treatment. Concerning the link between periodontitis and adverse pregnancy outcomes, almost half of the dentists reported being aware of this link mainly for pre-eclampsia (42.5%) and for low-birth weight of the child (49%). More than 2/3 of the dentists did not agree that the lack of time is the main reason for not giving enough information to pregnant women during counseling (77%).

**Table 2:** Knowledge and attitudes on perinatal oral health items

Knowledge/attitude	Agree	Disagree	Not sure
The link bewteen periodontitis and adverse birth outcome is not evident enough to inform pregnant women about it			
French-speaking (n=100) German-speaking (n=100) Total (n=200)	4 (4%) 3 (3%) 7 (3.5%)	61 (61%) 69 (69%) 130 (65%)	35 (35%) 28 (28%) 62 (31%)
Pregnant women should receive preventive dental care before and during pregnancy			
French-speaking (n=100) German-speaking (n=100) Total (n=200)	82 (82%) 83 (83%) 165 (82.5%)	2 (2%) 0 (0% 2 (1%)	16 (16%) 16 (16%) 32 (16%)
Pregnant women should only receive emergency dental care			
French-speaking (n=100) German-speaking (n=100) Total (n=200)	12 (12%) 12 (12%) 24 (12%)	36 (36%) 48 (48%) 92 (46%)	52 (52%) 40 (40%) 92 (42%)
Periodontitis during pregnancy increases the risk of pre-eclampsia			
French-speaking (n=100) German-speaking (n=100) Total (n=200)	39 (39%) 46 (46%) 85 (42.5)	13 (13%) 2 (2%) 15 (7.5%)	42 (42%) 42 (42%) 84 (42%)
Periodontitis during pregnancy increases the risk of low-birth weight baby			
French-speaking (n=100) German-speaking (n=100) Total (n=200)	47 (47%) 51 (51%) 98 (49%)	22 (22%) 5 (5%) 27 (13.5)	25 (25%) 41 (41%) 66 (33%)
Dental care should be part of prenatal care			
French-speaking (n=100) German-speaking (n=100) Total (n=200)	67 (67%) 95 (95%) 162 (81%)	7 (7%) 2 (2%) 9 (4.5%)	23 (23%) 3 (3%) 26 (13%)
I do not routinely advise pregnant women on oral health because it is time-consuming			
French-speaking (n=100) German-speaking (n=100) Total (n=200)	3 (3%) 6 (6%) 9 (4.5%)	79 (79%) 75 (75%) 154 (77%)	17 (17%) 18 (18%) 35 (17.5%)

Answers to questions about the appropriate timing for providing routine dental services to pregnant patients are shown in Table 3. In terms of radiographs, a majority of dentists (66% from the French part and 68% from the German part) think that they should be taken after delivery, whereas the remaining reported that the 2nd and 3rd trimesters are also safe. For supragingival cleaning, root planing, endodontic, prosthetic and orthodontic treatment, the timing was not considered important: the majority of the dentists responded that these measures can be performed at any time during pregnancy. For extractions, 47% of the dentists responded that any time during pregnancy is safe, but 40% of them replied that extractions should be performed after delivery. For surgery and tooth whitening, the most appropriate time was found to be after delivery by 61.5% and 74%, respectively. As second choice for most of the treatments, the 2nd trimester was reported to be the most appropriate time.

**Table 3:** Timing of safely conducting the following procedures during pregnancy by French- (n=100) and German-speaking (n=100) dentists

	1st trimester	2nd trimester	3rd trimester	Not important	After delivery	Don’t know
Radiographs						
French German	2 (2%) 0 (0%)	20 (20%) 11 (11%)	13 (13%) 17 (17%)	24 (24%) 22 (22%)	66 (66%) 68 (68%)	2 (2%) 3 (3%)
Supragingival scaling						
French German	4 (4%) 11 (11%)	15 (15%) 16 (16%)	5 (5%) 12 (12%)	78 (78%) 84 (84%)	16 (16%) 22 (22%)	2 (2%) 1 (1%)
Root planing						
French German	7 (7%) 6 (6%)	21 (21%) 15 (15%)	6 (6%) 8 (8%)	40 (40%) 45 (45%)	45 (45%) 45 (45%)	3 (3%) 4 (4%)
Extractions						
French German	7 (7%) 4 (4%)	27 (27%) 15 (15%)	7 (7%) 8 (8%)	32 (32%) 52 (52%)	40 (40%) 41 (41%)	8 (8%) 2 (2%)
Endodontics						
French German	1 (1%) 6 (6%)	19 (19%) 16 (16%)	7 (7%) 7 (7%)	52 (52%) 56 (56%)	34 (34%) 31 (31%)	2 (2%) 4 (4%)
Surgery						
French German	6 (6%) 1 (1%)	17 (17%) 9 (9%)	4 (4%) 4 (4%)	19 (19%) 37 (37%)	65 (65%) 58 (58%)	3 (3%) 5 (5%)
Fixed prosthesis						
French German	4 (4%) 6 (6%)	14 (14%) 11 (11%)	4(4%) 3 (3%)	52 (52%) 45 (45%)	39 (39%) 50 (50%)	5 (5%) 3 (3%)
Removable prosthesis						
French German	4 (4%) 5 (5%)	7 (7%) 10 (10%)	2 (2%) 5 (5%)	64 (64%) 66 (66%)	32 (32%) 37 (37%)	4 (4%) 1 (1%)
Orthodontics						
French German	3 (3%) 3 (3%)	5 (5%) 4 (4%)	2 (2%) 3 (3%)	47 (47%) 57 (57%)	50 (50%) 41 (41%)	5 (5%) 2 (2%)
Whitening						
French German	0 (0%) 2 (2%)	4 (4%) 3 (3%)	1 (1%) 5 (5%)	15 (15%) 29 (29%)	81 (81%) 67 (67%)	4 (4%) 4 (4%)

Local anesthesia was reported to be safe by 69% of both the French- and German-speaking dentists (Table 4). The remaining dentists estimated that anesthesia is contra-indicated during the first and third trimester (14% and 15%, respectively). The main reason as reported by only a minority of dentists was the risk for uterine contractions (6.5%) and premature delivery (10%). Anesthesia can be used with or without vasoconstriction (58% and 42%, respectively) and the injected compound used was reported to be articaine (30.5%), followed by lidocaine (12.5%) and merivacaine (10%).

**Table 4:** Administration of anesthetics and other drugs during pregnancy

Question 1. Is local anesthesia contraindicated during pregnancy?			
	French-speaking (n=100)	German-speaking (n=100)	Total (n=200)
No	69 (69%)	69 (69%)	138 (69%)
Yes, during the 1st trimester	15 (15%)	13 (13%)	28 (14%)
Yes, during the 2nd trimester	2 (2%)	2 (2%)	4 (2%)
Yes, during the 3rd trimester	14 (14%)	16 (16%)	30 (15%)
Question 2. Is the administration of antibiotics contraindicated during pregnancy?			
	French-speaking (n=100)	German-speaking (n=100)	Total (n=200)
No	33 (33%)	30 (30%)	63 (31.5%)
Yes, during the 1st trimester	55 (55%)	59 (59%)	114 (57%)
Yes, during the 2nd trimester	35 (35%)	37 (37%)	72 (36%)
Yes, during the 3rd trimester	38 (38%)	36 (36%)	74 (37%)
No answer	3 (3%)	2 (2%)	5 (2.5%)
Question 3. Is the administration of non-steroid anti-inflammatory drugs contraindicated during pregnancy?			
	French-speaking (n=100)	German-speaking (n=100)	Total (n=200)
No	10 (10%)	18 (18%)	28 (14%)
Yes, during the 1st trimester	64 (64%)	62 (62%)	126 (63%)
Yes, during the 2nd trimester	52 (52%)	29 (29%)	81 (40,5%)
Yes, during the 3rd trimester	65 (65%)	50 (50%)	115 (57,5%)
No answer	7 (7%)	5 (5%)	12 (6%)

One-third of the dentists reported that the administration of antibiotics is not a risk factor during pregnancy, 57% of the dentists reported that antibiotics should be avoided only during the first trimester, whereas the remaining dentists reported that during both the 2nd and 3rd trimesters antibiotics should not be prescribed (36% and 37%, respectively). The answers did not differ bewteen French- and German-speaking dentists. A large proportion of participants never prescribe metronidazole, macrolides, and doxycycline (53%, 52.5%, and 74.5%, respectively). Penicillin is the most frequently prescribed antibiotic. Finally, only a small proportion of dentists reported that non-steroidal anti-inflammatory drugs (NSAID) are safe during pregnancy. The majority found that the administration of NSAID should not be recommended during any trimester.

Concerning oral manifestations during pregnancy, dentists report that the most common by far is pregnancy gingivitis (77% and 87% for the French- and German-speaking parts, respectively). Caries, periodontal disease, mucosal lesions and tooth sensitivity were reported with much lower frequency. The main reason for pregnant women to consult their dentist is a routine check-up (52% and 67%), followed by gingival bleeding (17%) or due to an emergency (19.5%) (Table 5). The patients’ lack of knowledge for oral care during pregnancy as well as the lack of specific guidelines were perceived as the main barriers by dentists to providing dental care. Dentists from both parts of Switzerland (86% and 81%) reported the need for clear guidelines and direction on dental care during pregnancy.

**Table 5:** Reasons for and obstacles to dental care counseling during pregnancy

Question 1. Which are the most frequent oral manifestations during pregnancy?			
	French-speaking (n=100)	German-speaking (n=100)	Total (n=200)
Pregnancy gingivitis	77 (77%)	87 (87%)	164 (82.2%)
Caries	7 (7%)	5 (5%)	12 (6%)
Periodontitis	4 (4%)	2 (2%)	6 (3%)
Mucosal lesions	5 (5%)	4 (4%)	9 (4.5%)
Tooth sensitivity	5 (5%)	2 (2%)	7 (3.5%)
Erosions	1 (1%)	0 (0%)	1 (0.5%)
Question 2. Which are the main reasons for women to consult their dentist during pregnancy?			
	French-speaking (n=100)	German-speaking (n=100)	Total (n=200)
Gingival bleeding	19 (19%)	15 (15%)	34 (17%)
Infection	25 (25%)	14 (14%)	39 (19.5%
Routine check-up	52 (52%)	67 (67%)	119 (59.5%)
Mucosal lesions	2 (2%)	2 (2%)	4 (2%)
Others	2 (2%)	2 (2%)	4 (2%)
Question 3. Which are the main barriers to providing dental care for pregnant women?			
	French-speaking (n=100)	German-speaking (n=100)	Total (n=200)
Lack of practice guidelines on this topic	51 (51%)	30 (30%)	81 (40.5%)
Lack of time during counseling	4 (4%)	8 (8%)	12 (6%)
My lack of knowledge	67 (67%)	79 (79%)	146 (73%)
Patient’s lack of knowledge for oral care during pregnancy	48 (48%)	44 (44%)	92 (46%)
Other reasons	9 (9%)	3 (3%)	12 (6%)
Question 4. In your practice, do you need clear guidelines and direction on dental care during pregnancy?			
	French-speaking (n=100)	German-speaking (n=100)	Total (n=200)
Yes	86 (86%)	83 (83%)	169 (84.5%)
No	13 (13%)	14 (14%)	27 (13.5%)
No answer	1 (1%)	3 (3%)	4 (2%)

## Discussion

The aim of the present cross-sectional survey was to explore the knowledge and attitudes of a sample of Swiss dentists related to oral health practices for women during pregnancy. Although the questionnaire was sent to almost all parts of Switzerland, the majority of answers came from Geneva (the largest French-speaking Swiss city) and Zurich (the largest German-speaking Swiss city).

In general, few discrepancies were found between the 2 regions concerning dental care attitudes and practices towards pregnant women. Although education/training on dental care during pregnancy was provided to all dentists, only half of them estimated that it was sufficient. The majority of dentists emphasised the need for clear guidelines and direction on dental care during pregnancy.

The need for more knowledge was reported in various surveys addressed to gynaecologists,^18,33^ dental hygienists^2^ and general dentists.^5,8,13,22^

In our survey, almost all dentists agreed that pregnant women should receive preventive dental care before and during pregnancy and that dental care should be part of prenatal care. Positive attitudes towards delivering preventive care to women during pregnancy have been reported in several surveys.^10,39,42^ In our study, a small percentage of dentists (12%) estimated that pregnant women should only receive emergency dental care, whereas a substantial number of dentists (56% from the French-speaking part and 42% from the German-speaking) were not sure if dental care should be provided only when there is an emergency. Uncertainity was also reported on the link between periodontal disease and adverse pregnancy outcomes: less than half of the dentists believe that periodotitis increases the risk for pre-eclampsia and low-birth weight babies, and are thus not sure whether it is important to inform their patients. Interestingly, in a cross-sectional study performed with French gynaecologists and obstetricians,^7^ 88% were aware that periodontitis is an inflammatory disease that can negatively affect pregnancy outcomes. Half of them discussed this issue with their pregnant patients, but only one-third of them systemically referred the patient to a dentist. The authors concluded that although periodontal knowledge among French gynaecologists/obstetricians was satisfactory, their clinical behavior did not correlate with their knowledge.^7^

A recent concise review^31^ aimed to summarise the epidemiological findings and critically review the available data on the systemic effects of periodontitis, and more specifically on the link between periodontitis and adverse pregnancy outcomes. Based on the available epidemiological data ‘maternal periodontitis is modestly but significantly associated with preterm birth, low birth weight and preeclampsia’.^21^ However, as previously reported, interventional studies during the second trimester for treatment of maternal periodontal disease failed to demonstrate an improvement of gestational outcome.

Concerning the timing for safely conducting different procedures during pregnancy, very few participants reported not knowing (2-5% depending on the procedure). For the majority of the procedures, answers were equally distributed between ‘after delivery’ and ‘not important’. The third choice was ‘the second trimester’. The dentists indicated that radiographs, surgery and tooth whitening should be conducted after delivery. Scaling and root planing were considered safe by almost half of the participants (42%), and the other half suggested that it should be conducted after delivery. Similar surveys conducted in Oregon, USA, and Bangalore, India, reported that the the best timing for conducting routine care during pregnancy is the second trimester.^20,33^ Radiographs were the only procedure for which reponders’ attitudes differed: some authors reported that obtaining radiographs may be considered safe at any time during pregnancy ‘as long as the dental team follows basic guidelines of radiation exposure’, whereas others reported that they should be avoided during pregnancy unless absolutely necessary.^34^ Concerning the administration of anesthetics, the majority of the dentists (69%) reported that local anesthesia is not contra-indicated during pregnancy, whereas for antibiotics and non-steroiod antiinflammatory drugs, the opinions differed. For the latter, only a minority did not answer (2.5% and 6%, respectively).

Focusing on dentists, general practicioners, midwives and gynaecologists, a recent review by George et al^13^ examined their knowledge of and attitudes and behavior toward oral health care during pregnancy. The authors conluded that no real consensus existed among dentists and the other health practicioners on oral health during pregnancy. Many practicioners, including dentists, believe that dental procedures are not safe during pregnancy. Lack of knowledge was the main barrier reported by all practicioners to providing oral care in pregnancy. This was also the case in our study, where 73% of the participants confirmed this, in addition to the lack of specific guidelines on the topic (40.5%).

A recent systematic review examined the impact of existing oral health promotion interventions during pregnancy^40^ and reported a significant lack of oral health intervention programmes despite clear evidence and awareness among dentists on the link bewteen oral and systemic health.

Specific guidelines on dental care during pregnancy have been published,^37,41^ and although training is provided in terms of continuing education,^9^ still many dental professionals report difficulty in assessing the safety and correct timing of various dental procedures during pregnancy.

Our study showed that both French- and German-speaking dentists have received education on dental care during pregnancy, but this was deemed insufficient. Lack of knowledge and uncertainty towards several items on perinatal oral health were reported as the main barriers to providing dental care to pregnant women. The majority of the dentists expressed the need for clearer guidelines and direction regarding how and when to treat women during pregnancy.

## Conclusion

The findings from this survey suggest that Swiss dentists in private practice have the knowledge to provide dental care to pregnant women. Relatively small variations in knowledge were reported between the French- and German-speaking dentists. All dentists supported the need for evidence-based guidelines on this important aspect of public health.
